# Can preoperative percutaneous injection of ultrasound contrast agent locate sentinel lymph nodes of breast cancer?

**DOI:** 10.3389/fonc.2024.1471443

**Published:** 2024-11-25

**Authors:** Dayan Huang, Wenbin Cao, Yunhao Luo, Cheng Guan, Yuyan Liu, Chaonan Li, Jie Chen, Jing Luo, Jun Luo

**Affiliations:** ^1^ School of Medicine, University of Electronic Science and Technology of China, Chengdu, China; ^2^ Department of Ultrasound, Sichuan Provincial People's Hospital, University of Electronic Science and Technology of China, Chengdu, China; ^3^ School of Medical and Life Sciences, Chengdu University of Traditional Chinese Medicine, Chengdu, China; ^4^ Department of Breast Surgery, Sichuan Provincial People's Hospital, University of Electronic Science and Technology of China, Chengdu, China

**Keywords:** breast cancer, contrast-enhanced ultrasound, sentinel lymph node, marker, nanocarbon

## Abstract

**Objectives:**

We evaluated the ability and accuracy of preoperative identification and localization of sentinel lymph nodes (SLNs) using intradermal injection of ultrasound contrast agent.

**Materials and methods:**

Prospectively recruited 191 early breast cancer patients with clinically negative axillary lymph nodes (ALNs). All participants received intradermal injection of microbubble contrast agent. Following the identification and localization of SLNs using contrast-enhanced ultrasound (CEUS), Markers were deployed in the SLNs US-guided. Subsequently, the SLNs with Markers were stained and marked with a suspension of nano-carbon US-guided to assist in intraoperative localization of SLNs. Standard SLNB with methylene blue tracing was performed intraoperatively to assess the consistency between the two methods of SLNs localization, thereby determining the ability and accuracy of CEUS in identifying and localizing SLNs.

**Results:**

A total of 179 patients were included in the final evaluation analysis, in which a microbubble contrast agent was injected subcutaneously in the areolar region. A total of 201 SLNs were identified, with a median of 1 SLN per patient. Each SLN was identified in 157 patients, and two SLNs were identified in 22 patients. Among the 201 SLNs from the 179 patients, the proportion that could be individually matched between CEUS and the blue dye method was 95.5% (192/201), and the consistency evaluation in SLNs identification between CEUS and blue dye staining was excellent (Kappa value = 0.62, P < 0.001).

**Conclusion:**

The consistency of identification and localization of SLNs in early breast cancer patients between CEUS and the blue dye method was strong.

## Introduction

Breast cancer has become the most common malignant tumor in women worldwide, with approximately 2.31 million new diagnosed cases and 665,700 deaths in 2022 (1). The status of axillary lymph nodes (ALNs) is highly correlated with treatment strategies, disease-free survival, and overall survival rates of patients. Compared to patients with negative ALNs, those with ALN metastasis can experience up to a 40% decrease in 5-year overall survival rates (2). Sentinel lymph node biopsy (SLNB) is the standard procedure for axillary staging in early breast cancer patients with clinically negative ALNs (3–5).

The suitable method for sentinel lymph node (SLN) tracing plays a critical role in SLNB, facilitating accurate intraoperative localization of SLNs, enhancing the detection rate of SLNs, reducing the false-negative rate, and minimizing axillary damage and operative duration for patients. The preferred method for SLN mapping remains the dual-tracer technique using a combination of radioactive isotopes and blue dye in accordance with the standards of SCI medical journals (6, 7). However, the clinical applicability of the dual-tracer technique is limited. For example, in a survey of 110 large and medium-sized hospitals in China in 2018, only 15.5% of hospitals used or combined isotopic tracers (8).

Contrast-enhanced ultrasound (CEUS) of SLNs is an imaging technique that involves the percutaneous injection of contrast agents to visualize lymphatic channels and draining lymph nodes (LNs) in real-time. Previous studies (9–11) have observed the use of CEUS as a novel tracer for identifying and locating SLNs, but these studies applied the method of using a guide wire to mark the located SLNs to assist in intraoperative SLN identification. Due to the risk of guide wire dislodgement preoperatively or intraoperatively (12), precise marking of the identified SLNs using CEUS may not be feasible, making it challenging to correlate the CEUS-identified SLNs with the dissected LNs intraoperatively. Therefore, the controversy remains regarding whether the SLNs identified and located by CEUS are truly representative of the SLNs.

In this study, we applied US-guided placement of Markers and injection of nano-carbon suspension for dual labeling of SLNs, ensuring intraoperative examination of LNs matched one by one with the LNs identified and located preoperatively by CEUS. This was compared with the classic blue dye method to evaluate the accuracy of preoperative identification and localization of SLNs using percutaneous CEUS.

## Materials and methods

### Patient selection

**Figure 1:**
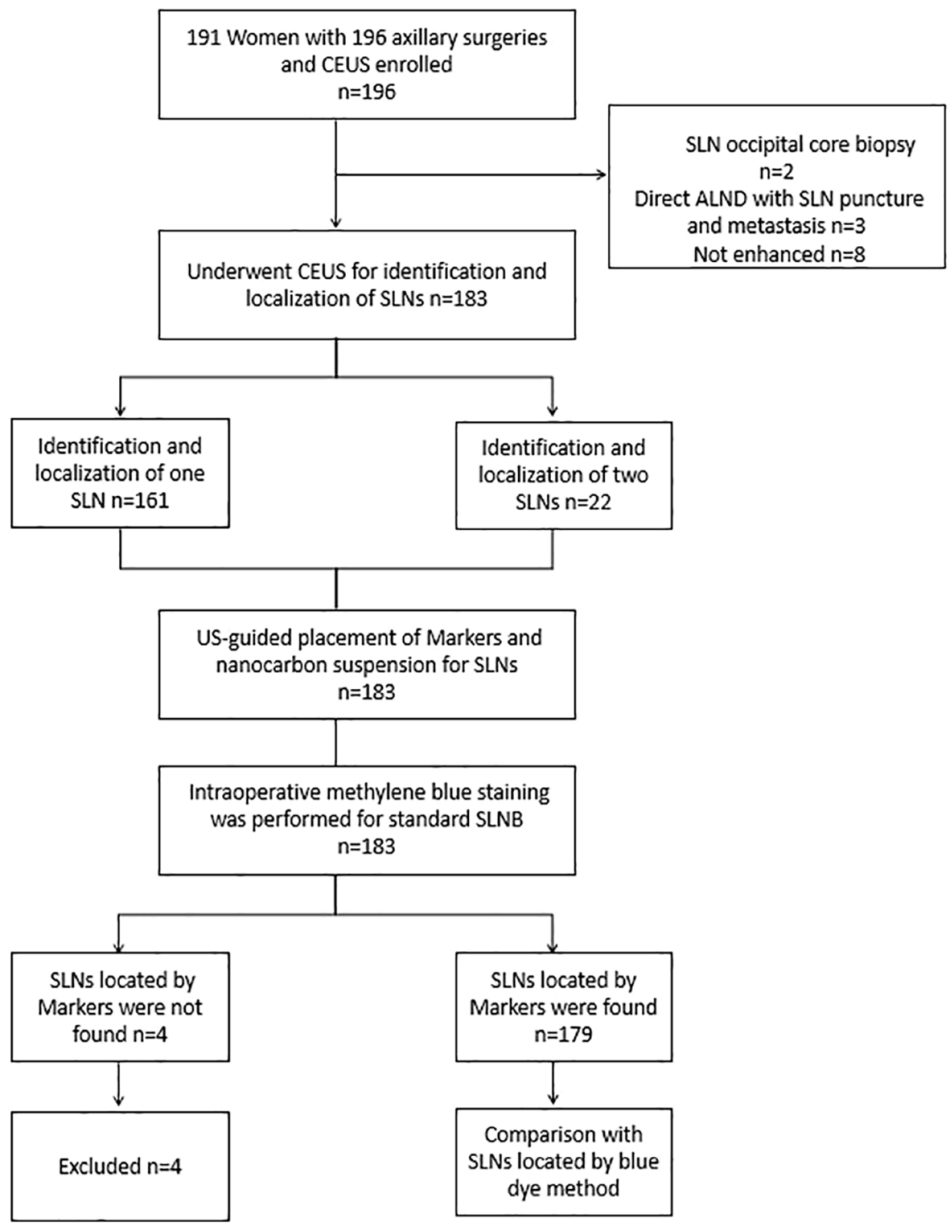
Flowchart shows the basic procedure of the study was presented. CEUS, contrast-enhanced ultrasound; SLN, sentinel lymph node; SLNB, sentinel lymph node biopsy; ALND, axillary lymph node dissection.

This study was approved by the institutional review board and ethics committee of Sichuan Provincial People’sHospital, Batch Number: Ethics Review (Research) No. 261, 2022. The method of CEUS and Marker placement under guidance was conducted according to the approved guidelines, and written informed consent was obtained from all participants before registration. From February 6, 2023, to March 1, 2024, a total of 191 patients were prospectively recruited for the study, with the study flowchart shown in **Figure 1**. Inclusion criteria included: (1) histologically confirmed breast cancer through core needle biopsy or local resection, (2) clinically assessed negative ALNs, (3) patients undergoing SLNB, (4) ultrasound contrast-enhanced imaging-guided identification and placement of Markers in the SLNs, followed by the injection of diluted nano-carbon the day before surgery. Exclusion criteria included: (1) pregnant or breastfeeding women, (2) inflammatory breast cancer, (3) allergy to ultrasound contrast agents, (4) previous ALN dissection; (5) severe internal and surgical comorbidities.

### Conventional US and CEUS examination

On the day prior to surgery, patients underwent ultrasonography of the ipsilateral ALNs with the same body position as during the surgical procedure, to reassess for suspicious sonographic signs of ALN involvement. Patients with suspected abnormal ALN were excluded. Following the conventional US examination, CEUS was performed by injecting microbubble contrast agent (SonoVue, mixed with 5ml of sterile saline water, with each point was injected with 0.5 ml.) subcutaneously around the areola at the 3/6/9/12 o’clock positions of the breast. Subsequent gentle massage was applied to facilitate the entry of microbubble into the lymphatic vessels and ALNs. The contrast mode was then used to track the enhanced lymphatic vessels and their drainage to the LNs, to identify the enhanced lymphatic vessels and draining LNs of the affected breast. The first enhanced node was deemed the SLN with enhancement. Real-time dual imaging was then conducted to confirm the presence of structurally defined LNs. Once a clear LN structure was identified on the grayscale image, the SLN could be localized. If the lymphatic vessels were not clearly visualized, after subcutaneous and intradermal supplementary injection of 0.5-1.0 ml microbubble contrast agent around the tumor, observation was conducted.

### US-guided placement of Markers and nano-carbon suspension for SLNs

**Figure 2:**
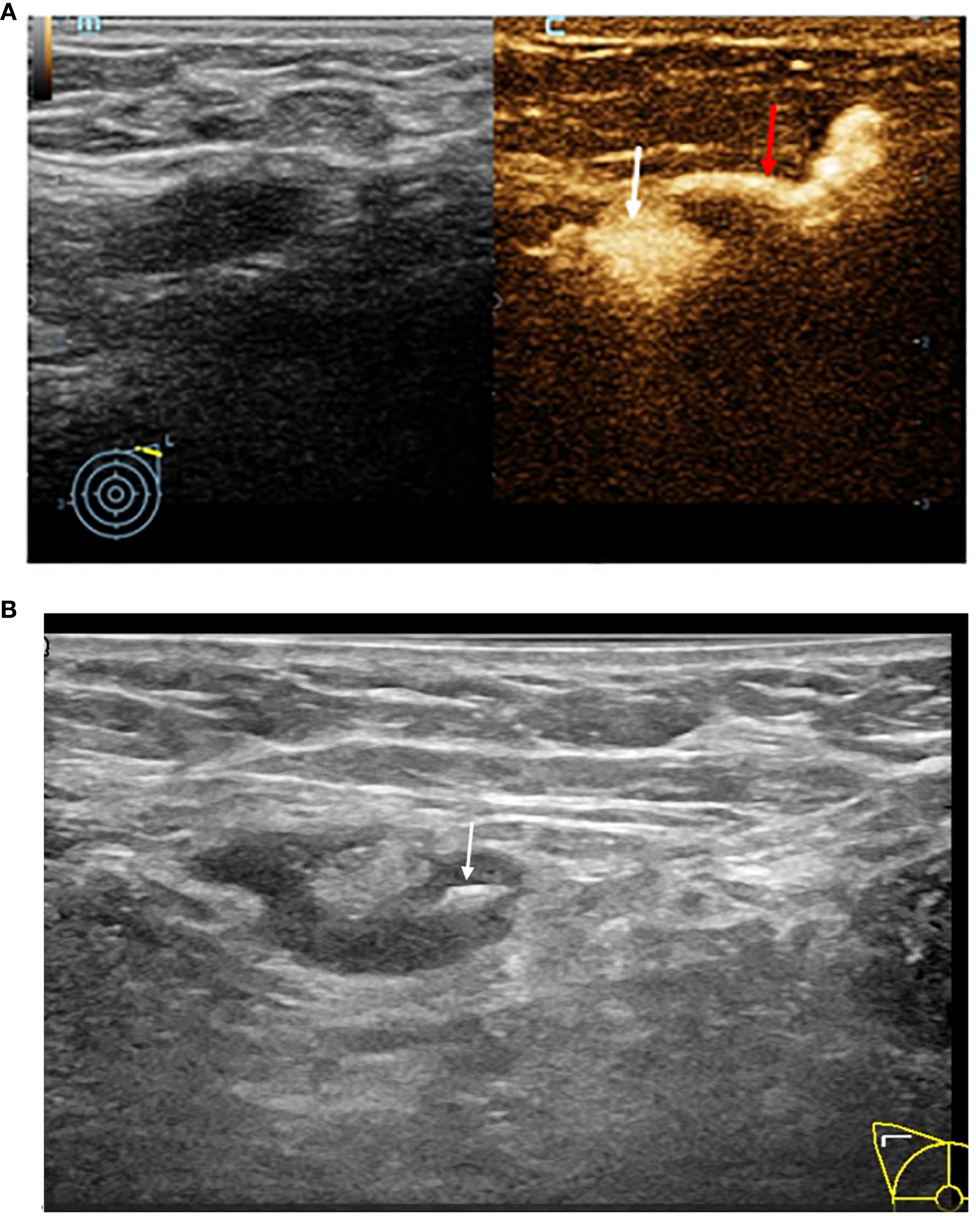
**(A)** This image demonstrates the typical accumulation of contrast agent within the lymph node structure, contrast pulse sequencing image of an afferent lymphatic vessel (red arrow) entering a SLN (white arrow). **(B)** Grey-scale image of a SLN being localized by a hyper-echoic Marker (white arrow).

The patients first underwent routine axillary disinfection and local anesthesia. Then, the Marker(Bard 864017D) was placed with real-time ultrasound guidance within the cortex of the SLN. After the Marker was inserted, a gray-scale US examination was performed to confirm its position, which appeared as a high-echo (**Figure 2**). Subsequently, 0.5ml of diluted nano-carbon suspension was injected with US guidance onto the cortical surface of the SLN where the Marker was placed to assist in accurately locating that during SLNB. The 1ml of diluted nano-carbon suspension was prepared by mixing 0.1ml of nano-carbon suspension with sterile saline at a 1:10 ratio to ensure that the regional LNs were not excessively stained. Finally, the locations of the SLNs and the courses of the draining lymphatic vessels on the body surface were marked to guide the selection of the SLNB surgical incision and intraoperative identification of the SLNs. If multiple SLNs were identified with CEUS, all detected SLNs were marked with a Marker and stained with nano-carbon under ultrasound guidance. The size, number, location, distance from the body surface, enhancement pattern of the SLNs, the position and number of lymphatic vessels, and the number of Markers placed were recorded. All the aforementioned procedures were completed within 15 minutes.

### Intraoperative specimen dissection

**Figure 3:**
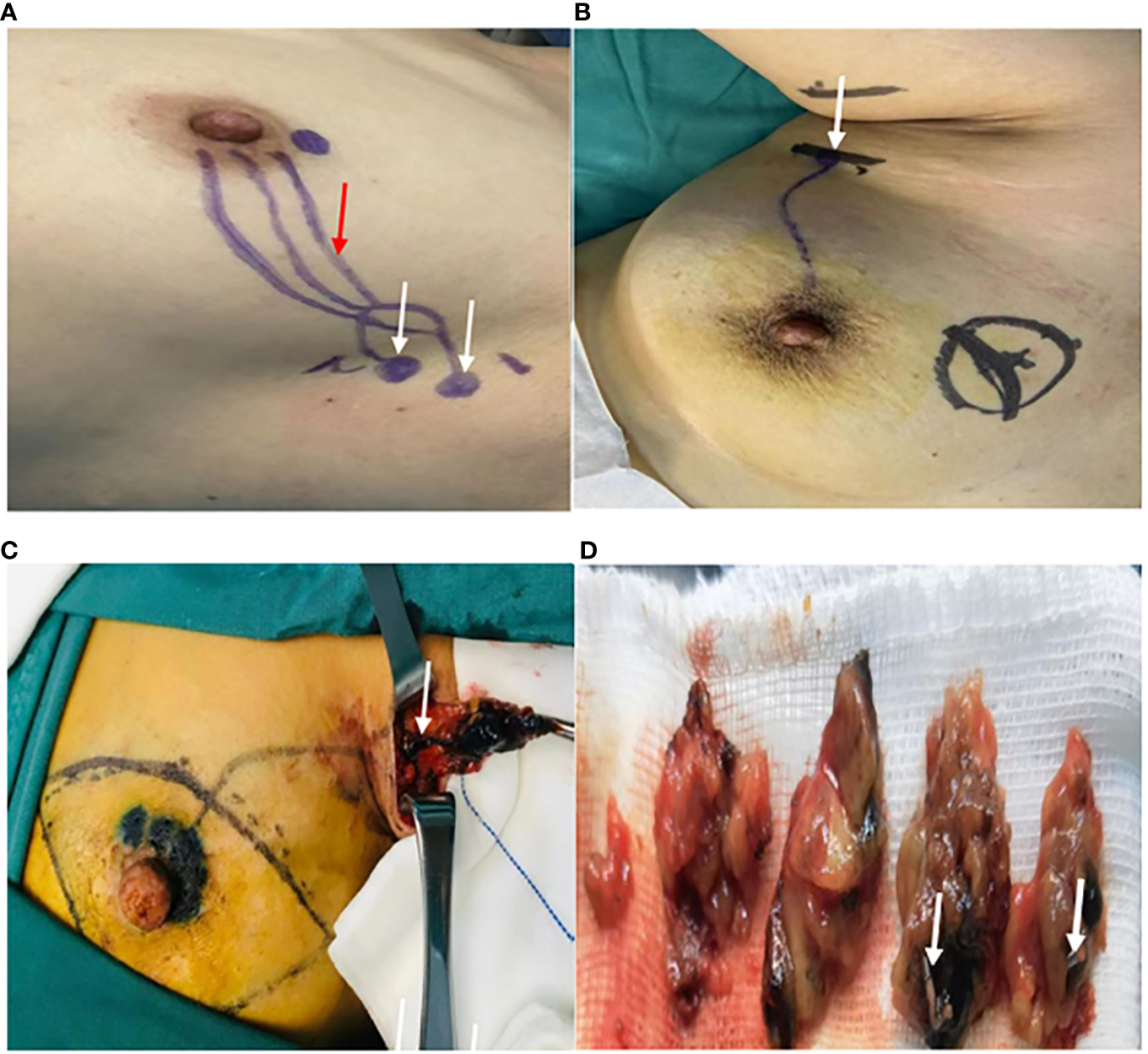
Searching for the SLNs during the surgery. **(A)** The location of the SLNs (white arrow) and the course of the draining lymphatic vessels(red arrow) on the body surface being marked as skin Markers. **(B)** The axillary incision (white arrow) being selected based on preoperative surface localization. **(C)** The first stained lymph node (white arrow) found along the blue-dyed lymphatic vessel. **(D)** The excision of the cut-open SLNs localized by the Markers (white arrow).

According to the standard procedure of SLNB, 2 ml of methylene blue was injected intradermally in the periareolar region 10-15 minutes preoperatively, similar to the injection method of the microbubble contrast agent described earlier. Intraoperatively, the axillary incision was selected based on preoperative surface localization, and all first-station stained LNs along the blue-dyed lymphatic vessels were identified as SLNs. The consistency between the LNs located by blue dye and nano-carbon suspension was observed. SLNs located by blue dye were excised and dissected to check for the presence of a Marker inside. The presence of a Marker indicated concordance between the SLNs identified by CEUS and blue dye. The intraoperative SLN identification process is shown in **Figure 3**. All blue-dyed SLNs, Marker-labeled SLNs, and other ALNs in the axilla were individually marked, subjected to intraoperative rapid frozen section examination, and routine postoperative pathological examination. The comparison between CEUS-located SLNs and blue dye-located SLNs during biopsy, the number of SLNs determined intraoperatively, the number of LNs sent for examination, and pathological results were recorded.

### Statistical analysis

In this study, data analysis was conducted using SPSS 25.0 (SPSS Inc., Chicago, IL, USA). The consistency of identifying SLNs using percutaneous CEUS and blue dye method was evaluated with Kappa test. The accuracy of percutaneous CEUS in identifying and locating SLNs was assessed by comparing the results with those determined by the blue dye method. P < 0.05 was considered to indicate a statistically significant difference.

## Results

### Patient characteristics

A total of 196 SLNBs from 191 patients were included in the evaluation. Among them, 5 patients underwent bilateral SLNB. This study analyzed one patient who underwent SLNB procedure on one side as one case. Two cases only underwent US-guided biopsy, while in 3 cases where metastases were found during the puncture biopsy, ALND was performed directly. Among the remaining 191 patients, 95.8% (183/191) showed enhancement of SLNs after CEUS. SLNs were all placed and injected with nano-carbon suspension under US-guidance, and Marker was successfully found intraoperatively in 179 patients. In the final analysis of these 179 patients, the median age was 50 years (range 22-89 years), all of whom were pathologically confirmed to have early breast cancer. Patient and tumor characteristics are detailed in **Table 1**.

**Table 1:** Baseline patient and tumor characteristics.

Baseline characteristics	Number of Patients (n=179)
Age (years)	50
Laterality of SLNB	
Unilateral	169
Bilateral	5
Orientation	
Upper outer quadrant	77
Upper inner quadrant	43
Lower outer quadrant	33
Lower inner quadrant	16
Central quadrant	7
Multifocal	3
Clinical T category	
cTis	23
cT1	90
cT2	58
cT3	6
cT4	2
Histological type	
DCIS	25
IDC	142
Other	12
Tumor subtype	
Luminal B	75
Luminal A	47
Basal-like	25
ERBB2+	19
Not available	13
Surgical procedure	
SLNB	156
SLNB+ALND	23
**Total**	179

SLNB, sentinel lymph node biopsy; ALND, axillary lymph node dissection; DCIS, ductal carcinoma in situ; IDC, invasive ductal carcinoma.

### Identification of SLNs

Subcutaneous injection of the microbubble contrast agent in the areola area did not visualize the LNs in 8 cases, resulting in an overall identification rate of 95.8% (183/191). One SLN was identified in 161 patients, while two SLNs were identified in 22 cases. In total, 205 SLN Markers were placed in 183 patients, with 4 Markers not found in 4 patients during SLNB, resulting in the loss rate of Markers was 2% (4/205), and Markers were successfully found intraoperatively in 179 patients.

A total of 768 ALNs were detected in 179 patients using the methylene blue method, among which 210 were SLNs. The median number of SLNs per patient was 1 (mean: 1.2). After subcutaneous injection of the microbubble contrast agent in the areola area, 201 SLNs were detected in 179 patients, with a median of 1 SLN per patient (mean: 1.1), the specific results are shown in **Table 2**. Among the 201 SLNs in 179 patients, the correspondence rate between CEUS and the blue dye method was 95.5% (192/201), with a Kappa value of 0.62 for the consistency of SLN identification between CEUS and the blue dye method (P < 0.001), the specific results are detailed in **Table 3**.

**Table 2:** The consistency of SLNs identification and localization between CEUS and blue dye method.

	Methylene Blue					
CEUS		1	2	3	4	K=0.62
	1	148	7	1	1	
	2	5	15	2	0	

SLN, sentinel lymph node; CEUS, contrast-enhanced ultrasound.

**Table 3:** The number of SLNs in the CEUS group and methylene blue group.

Group	n				
		1	2	3	4
CEUS	179	157	22	0	0
Methylene Blue	179	153	22	3	1

CEUS, contrast-enhanced ultrasound.

### Histopathological examination

Among the 179 patients included in the final analysis, forty cases were found to have metastases in SLNs. CEUS identified and located SLNs in 34 cases (85%), while the blue dye method located SLNs in 33 cases (82.5%). Among the 5 patients in whom CEUS identified and located 2 SLNs with metastases, the blue dye method failed to identify the second SLN in 2 cases, accounting for 40% (2/5). In contrast, among the 12 patients in whom the blue dye method identified and located multiple SLNs with metastases, only 1 patient had SLNs with metastases that were not identified by CEUS, accounting for 8% (1/12). The proportion of patients with SLNs containing metastases that were not identified by either CEUS or the blue dye method was 12.5% (5/40).

## Discussion

This prospective study confirmed that CEUS could accurately preoperatively locate SLNs of patients with earlybreast cancer. By preoperatively injecting the microbubble contrast agent percutaneously to enhance lymphatic vessels and thereby track and identify SLNs, a comparison was made with intraoperative blue dye staining. Among the 201 SLNs identified from 179 patients, the proportion of SLNs that could be matched one by one between CEUS and blue dye staining was 95.5%. CEUS and blue dye staining showed strong concordance in identifying SLNs, with a Kappa value of 0.62, confirming that the LNs located by CEUS were true SLNs. The majority of patients exhibited only one SLN on CEUS, aligning with previous studies (9–11, 13, 14), while 22 patients had two SLNs identified. In this study, compared to blue dye staining, CEUS-guided SLN localization exhibited a lower false-negative rate inbiopsy procedures. This underscores the importance of integrating various tracing techniques for accurate SLN biopsy and affirms that dual-tracing methods result in a lower false-negative rate compared to single-tracing methods. While the SLNs identified by CEUS and blue dye staining were consistent, the proportion of patients with identified metastases in SLNs that were not detected by either method was 12.5% (5/40). Consequently, in clinical practice, to reduce the false-negative rate of SLNB, multiple SLN tracing methods are usually combined to examine 3-6 LNs. This is one of the main reasons why the number of LNs examined in clinical practice is often exceeds the true number of SLNs (4, 15, 16).

The related studies (9–11) have applied CEUS for preoperative identification and localization of SLNs, and used guide wires to mark the SLNs. However, due to issues such as movement of the patient’s arm on the affected side after preoperative guide wire placement or intraoperative traction and anatomical dissection, there is a risk of guide wire slippage and displacement, which raises questions about whether the SLNs sent for pathological testing during surgery can be individually matched with the SLNs preoperatively located by CEUS. The application of US-guided placement of Markers for marking has been demonstrated to overcome the aforementioned difficulties. Due to the small size of the Markers, accurate preoperative localization ensures that the LNs submitted for examination are those identified and localized by CEUS. This method aids surgeons in accurately and comprehensively identifying the true SLNs, thereby reducing the incidence of false-negative rate. Compared with the blue dye method, this approach further confirms the localization capability of CEUS for SLNs. In this study, nano-carbon suspension was injected onto the cortical surface of the SLNs with Markers placed under US-guidance, facilitating the sensitive and clear identification of preoperatively localized SLNs by the naked eye during surgery. This method reduces the difficulty of finding stained LNs intraoperatively, shortenssurgery time, and increases the confidence ofsurgeons.

Currently, the preferred tracing technique for SLNB is the dual-tracing method combining radioactive isotopes with blue dye. However, the use of radioactive isotopes as tracers has certain limitations (17). The acquisition, storage, use, and disposal of radioactive isotopes require strict training, and concerns about radiation exposure limit the clinical applicability of SLNB guided by dual-tracing techniques involving radioactive isotopes. Injection of blue dye may increase the risk of complications due to excessive LNs excision and have a certain impact on the aesthetic appearance of the patient’s skin (18, 19). Importantly, using blue dye alone requires a high level of technical skill from surgeon, leading to lower success rates and higher false-negative rate (20). A study (21) has shown that the false-negative rate of SLNB performed using blue dye alone was 8.6%, higher than that of SLNB guided by combined tracing methods. Therefore, exploring the use of CEUS in combination with blue dye for SLN tracing and localization to reduce the false-negative rate of SLNB holds significant clinical value. The microbubble contrast agent used in CEUS have a higher relative molecular weight compared to blue dye. When using microbubble contrast agent to locate SLNs, the LNs detected within a few minutes post-drug injection are the first enhanced LNs (22–24). Additionally, the ultrasound contrast agent used in this study consists of phospholipid-encapsulated sulfur hexafluoride microbubbles, which is without protein components, thus reducing the occurrence of allergic reactions when using CEUS to identify and locate SLNs (25). Therefore, exploring CEUS combined with blue dye as an alternative dual-tracing method for SLNB offers certain advantages.

The commonly used dye method, fluorescence tracing method, and isotope tracing method in clinical practice (26–29) are all operational techniques performed in real-time during surgery. Prior to making the surgical incision, the successful localization of SLNs cannot be assessed, aand pinpointing the specific location of SLNs preoperatively is challenging. This often leads to the selection of SLNB incisions based on empirical methods. For patients with variable SLN locations, the difficulty of intraoperative search is frequently increased, which may potentially prolong the surgical duration and require the enlargement of the incision during the procedure. Therefore, preoperative precise localization and identification of SLNs are crucial for further evaluating the ALN status and devising more appropriate treatment plans. Due to its high temporal resolution, CEUS can dynamically display the enhancement process of draining lymphatic vessels and enhanced LNs in real-time after the injection of microbubble contrast agents. This study has confirmed its strong consistency with the blue dye method in identifying SLNs. Additionally, CEUS can preoperatively determine the success rate of SLN tracing and the anatomical location of the projected SLNs on the body surface, guiding the selection of surgical incisions, reducing the difficulty of intraoperative SLN search, boosting the confidence of the surgeon, and avoiding intraoperative SLNB failure due to the unsuccessful use of other tracing methods. Therefore, it can be used for preoperative localization and evaluation of SLNs in patients.

Although SLNB reduced the occurrence of postoperative complications compared to ALND, it is still an invasive procedure. Some scholars (30–32) have explored whether US-guided biopsy, as a minimally invasive method, can serve as an alternative to SLNB, transitioning SLNB from invasive to minimally invasive. Only through the identification and localization of SLNs by CEUS followed by US-guided biopsy targeted at SLN can this method become feasible. In this study, three patients underwent SLN core needle biopsy under ultrasound guidance following CEUS identification and localization, which confirmed metastasis. Consequently, these patients directly proceeded to ALND, thereby bypassing the time-consuming SLNB. With the advancement of medical technology and the increasing focus on personal health, patients and healthcare professionals are increasingly pursuing and recognizing precise, minimally invasive, and even non-invasive diagnostic and therapeutic methods. In the SOUND trial (33), all patients included in the study had negative results on preoperative axillary two-dimensional ultrasound examination. However, postoperative pathological results in the control group (SLNB group) revealed that 13.7% of patients still had ALN metastasis. This suggests that preoperative ultrasound evaluation of ALNs still has a certain false negative rate and is not sufficiently enough. Conventional ultrasound cannot determine whether the observed LNs are SLNs, and sometimes SLNs with atypical LN morphology are overlooked, leading examiners to broadly assess all identifiable ALNs in evaluating a patient’s ALN status, making it difficult to focus on a detailed assessment of solely the SLNs. Based on the identification and localization of SLNs by CEUS and the joint assessment with two-dimensional ultrasound imaging, can the diagnostic accuracy of ALNs in breast cancer patients be further improved preoperatively, reducing the false negative rate and allowing truly node-negative patients to be spared from biopsy, achieving precise axillary surgical management and transitioning from pathological N0 (pN0) to imaging N0 (iN0).

This study has several limitations. Firstly, as a single-center study with a relatively small number of patients, it would be beneficial to conduct larger multi-center studies with a larger patient cohort to validate the findings. Secondly, due to restrictions on the use of radioisotope, this study only compared the results with the blue dye method. To further assess the accuracy of SLN identification by CEUS, it would be necessary to compare it with the standard dual-tracer technique combining radioisotope and blue dye. Lastly, because the Z0011 trial (34) did not include ALND after SLNB, it was not possible to determine the false-negative rate of SLNB. Long-term follow-up is needed to observe the distant metastatic situation of the ALNs to provide a more comprehensive evaluation of the procedure’s accuracy and safety.

## Conclusion

This study confirmed a strong consistency in the identification and localization of SLNs in early breast cancer patients between CEUS and the blue dye method. Preoperative percutaneous injection of the ultrasound contrast agent can accurately identify and locate the SLNs of patients with early breast cancer. In the future, combining CEUS with other tracing methods for SLNs may reduce the false negative rate of SLNB, providing a non-radioactive alternative for dual tracing of SLNs.

## Data Availability

The raw data supporting the conclusions of this article will be made available by the authors, without undue reservation.

## References

[1] Bray F Laversanne M Sung H Ferlay J Siegel RL Soerjomataram I. Global cancer statistics 2022: GLOBOCAN estimates of incidence and mortality worldwide for 36 cancers in 185 countries. CA: Cancer J Clin. (2024) 74:229–63. doi: 10.3322/caac.21834 38572751

[2] Danko ME Bennett KM Zhai J Marks JR Olson JA Jr. Improved staging in node-positive breast cancer patients using lymph node ratio: results in 1,788 patients with long-term follow-up. J Am Coll Surgeons. (2010) 210:797–805. doi: 10.1016/j.jamcollsurg.2010.02.045 20421053

[3] Kim T Giuliano AE Lyman GH. Lymphatic mapping and sentinel lymph node biopsy in early-stage breast carcinoma: a metaanalysis. Cancer. (2006) 106:4–16. doi: 10.1002/cncr.v106:1 16329134

[4] Dn K. Technical outcomes of sentinel-lymph-node resection and conventional axillary-lymph-node dissection in patients with clinically node-negative breast cancer: results from the NSABP B-32 randomized phase III trial. Lancet Oncol. (2007) 8:881–88. doi: 10.1016/S1470-2045(07)70278-4 17851130

[5] Heerdt AS. Lymphatic mapping and sentinel lymph node biopsy for breast cancer. JAMA Oncol. (2018) 4:431. doi: 10.1001/jamaoncol.2017.4000 29167885

[6] Cody HS Fey J Akhurst T Fazzari M Mazumdar M Yeung H. Complementarity of blue dye and isotope in sentinel node localization for breast cancer: univariate and multivariate analysis of 966 procedures. Ann Surg Oncol. (2001) 8:13–9. doi: 10.1007/s10434-001-0013-9 11206218

[7] Tafra L Lannin DR Swanson MS Van Eyk JJ Verbanac KM Chua AN. Multicenter trial of sentinel node biopsy for breast cancer using both technetium sulfur colloid and isosulfan blue dye. Ann Surg. (2001) 233:51–9. doi: 10.1097/00000658-200101000-00009 PMC1421166 11141225

[8] Yang B Ren G Song E Pan D Zhang J Wang Y. Current status and factors influencing surgical options for breast cancer in China: a Nationwide cross-sectional survey of 110 hospitals. Oncol. (2020) 25:e1473–e80. doi: 10.1634/theoncologist.2020-0001 PMC7543333 32333626

[9] Xie F Zhang D Cheng L Yu L Yang L Tong F. Intradermal microbubbles and contrast-enhanced ultrasound (CEUS) is a feasible approach for sentinel lymph node identification in early-stage breast cancer. World J Surg Oncol. (2015) 13:1–8. doi: 10.1186/s12957-015-0736-x 26585236 PMC4653941

[10] Saidha NK Aggarwal R Sen A. Identification of sentinel lymph nodes using contrast-enhanced ultrasound in breast cancer. Indian J Surg Oncol. (2017) 9:355–61. doi: 10.1007/s13193-017-0646-1 PMC6154376 30287998

[11] Xu Y-L Liu X-J Zhu Y Lu H. Preoperative localization of sentinel lymph nodes using percutaneous contrast-enhanced ultrasonography in patients with breast cancer. Gland Surg. (2022) 11:369–77. doi: 10.21037/gs-22-10 PMC8899426 35284303

[12] Kapoor MM Patel MM Scoggins ME. The wire and beyond: recent advances in breast imaging preoperative needle localization. Radiographics. (2019) 39:1886–906. doi: 10.1148/rg.2019190041 31560614

[13] Sever A Jones S Cox K Weeks J Mills P Jones P. Preoperative localization of sentinel lymph nodes using intradermal microbubbles and contrast-enhanced ultrasonography in patients with breast cancer. J Br Surg. (2009) 96:1295–99. doi: 10.1002/bjs.6725 19847869

[14] Niu Z Gao Y Xiao M Mao F Zhou Y Zhu Q. Contrast-enhanced lymphatic US can improve the preoperative diagnostic performance for sentinel lymph nodes in early breast cancer. Eur Radiol. (2023) 33:1593–602. doi: 10.1007/s00330-022-09139-x PMC9510155 36152038

[15] Ban EJ Lee JS Koo JS Park S Kim SI Park B-W. How many sentinel lymph nodes are enough for accurate axillary staging in t1-2 breast cancer? J Breast Cancer. (2011) 14:296. doi: 10.4048/jbc.2011.14.4.296 22323916 PMC3268926

[16] Yi M Meric-Bernstam F Ross MI Akins JS Hwang RF Lucci A. How many sentinel lymph nodes are enough during sentinel lymph node dissection for breast cancer? Cancer. (2008) 113:30–7. doi: 10.1002/cncr.v113:1 PMC4365472 18457326

[17] Goonawardena J Yong C Law M. Use of indocyanine green fluorescence compared to radioisotope for sentinel lymph node biopsy in early-stage breast cancer: systematic review and meta-analysis. Am J Surg. (2020) 220:665–76. doi: 10.1016/j.amjsurg.2020.02.001 32115177

[18] Bakri NAC Kwasnicki RM Khan N Ghandour O Lee A Grant Y. Impact of axillary lymph node dissection and sentinel lymph node biopsy on upper limb morbidity in breast cancer patients: a systematic review and meta-analysis. Ann Surg. (2023) 277:572–80. doi: 10.1097/SLA.0000000000005671 PMC9994843 35946806

[19] Thevarajah S Huston TL Simmons RM. A comparison of the adverse reactions associated with isosulfan blue versus methylene blue dye in sentinel lymph node biopsy for breast cancer. Am J Surg. (2005) 189:236–39. doi: 10.1016/j.amjsurg.2004.06.042 15720998

[20] Li J Chen X Qi M Li Y. Sentinel lymph node biopsy mapped with methylene blue dye alone in patients with breast cancer: a systematic review and meta-analysis. PloS One. (2018) 13:e0204364. doi: 10.1371/journal.pone.0204364 30235340 PMC6147575

[21] Pesek S Ashikaga T Krag LE Krag D. The false-negative rate of sentinel node biopsy in patients with breast cancer: a meta-analysis. World J Surg. (2012) 36:2239–51. doi: 10.1007/s00268-012-1623-z PMC3469260 22569745

[22] Liu J Liu X He J Gou B Luo Y Deng S. Percutaneous contrast-enhanced ultrasound for localization and diagnosis of sentinel lymph node in early breast cancer. Sci Rep. (2019) 9:13545. doi: 10.1038/s41598-019-49736-3 31537856 PMC6753066

[23] Shimazu K Miyake T Tanei T Naoi Y Shimoda M Kagara N. Real-time visualization of lymphatic flow to sentinel lymph nodes by contrast-enhanced ultrasonography with sonazoid in patients with breast cancer. Ultrasound Med Biol. (2019) 45:2634–40. doi: 10.1016/j.ultrasmedbio.2019.07.005 31371127

[24] Miyake T Shimazu K Tanei T Naoi Y Kagara N Shimoda M. Hookwire-guided sentinel lymph node biopsy using contrast-enhanced ultrasonography followed by a one-step nucleic acid amplification (OSNA) Assay for Breast Cancer. Anticancer Res. (2019) 39:6183–92. doi: 10.21873/anticanres.13826 31704846

[25] Shang Y Xie X Luo Y Nie F Luo Y Jing X. Safety findings after intravenous administration of sulfur hexafluoride microbubbles to 463,434 examinations at 24 centers. Eur Radiol. (2023) 33:988–95. doi: 10.1007/s00330-022-09108-4 36205769

[26] Ahmed M Purushotham AD Douek M. Novel techniques for sentinel lymph node biopsy in breast cancer: a systematic review. Lancet Oncol. (2014) 15:e351–e62. doi: 10.1016/s1470-2045(13)70590-4 24988938

[27] Ballardini B Santoro L Sangalli C Gentilini O Renne G Lissidini G. The indocyanine green method is equivalent to the 99mTc-labeled radiotracer method for identifying the sentinel node in breast cancer: a concordance and validation study. Eur J Surg Oncol (EJSO). (2013) 39:1332–36. doi: 10.1016/j.ejso.2013.10.004 24184123

[28] Hirche C Murawa D Mohr Z Kneif S Hünerbein M. ICG fluorescence-guided sentinel node biopsy for axillary nodal staging in breast cancer. Breast Cancer Res Treat. (2010) 121:373–78. doi: 10.1007/s10549-010-0760-z 20140704

[29] Bargon CA Huibers A Young-Afat DA Jansen BA Borel-Rinkes IH Lavalaye J. Sentinel lymph node mapping in breast cancer patients through fluorescent imaging using indocyanine green: the INFLUENCE trial. Ann Surg. (2022) 276:913–20. doi: 10.1097/SLA.0000000000005633 35894448

[30] Zhong J Sun DS Wei W Liu X Liu J Wu X. Contrast-enhanced ultrasound-guided fine-needle aspiration for sentinel lymph node biopsy in early-stage breast cancer. Ultrasound Med Biol. (2018) 44:1371–78. doi: 10.1016/j.ultrasmedbio.2018.03.005 29631800

[31] Cox K Taylor-Phillips S Sharma N Weeks J Mills P Sever A. Enhanced pre-operative axillary staging using intradermal microbubbles and contrast-enhanced ultrasound to detect and biopsy sentinel lymph nodes in breast cancer: a potential replacement for axillary surgery. Br J Radiol. (2018) 91:20170626. doi: 10.1259/bjr.20170626 PMC5965773 29125333

[32] Zhou P Zheng W Liu Y Wang Y. Preoperative contrast-enhanced ultrasound (CEUS) combined with 125I seeds localization in sentinel lymph node biopsy for breast cancer. Cancer Manage Res. (2021) 13:1853–60. doi: 10.2147/CMAR.S296142 PMC7917323 33658849

[33] Gentilini OD Botteri E Sangalli C Galimberti V Porpiglia M Agresti R. Sentinel lymph node biopsy vs no axillary surgery in patients with small breast cancer and negative results on ultrasonography of axillary lymph nodes. JAMA Oncol. (2023) 9:1557–64. doi: 10.1001/jamaoncol.2023.3759 PMC10514873 37733364

[34] Giuliano AE Ballman KV McCall L Beitsch PD Brennan MB Kelemen PR. Effect of axillary dissection vs no axillary dissection on 10-year overall survival among women with invasive breast cancer and sentinel node metastasis: the ACOSOG Z0011 (Alliance) randomized clinical trial. Jama. (2017) 318:918–26. doi: 10.1001/jama.2017.11470 PMC5672806 28898379

